# Stoichiometry and Life-History Interact to Determine the Magnitude of Cross-Ecosystem Element and Biomass Fluxes

**DOI:** 10.3389/fmicb.2017.00814

**Published:** 2017-05-09

**Authors:** Thomas M. Luhring, John P. DeLong, Raymond D. Semlitsch

**Affiliations:** ^1^Biological Sciences, University of NebraskaLincoln, NE, USA; ^2^Biological Sciences, University of MissouriColumbia, MO, USA; ^3^Savannah River Ecology Laboratory, Ecology, University of GeorgiaAiken, SC, USA

**Keywords:** body size, calcium, carbon, nitrogen, nutrient cycling, phosphorus, reciprocal subsidy

## Abstract

Ecosystems are linked through the transfer of materials and energy. Studies examining material fluxes across habitat boundaries frequently quantify unidirectional flows of nutrients and energy. However, material fluxes can be multidirectional, and we lack a conceptual framework to describe how their quantity and stoichiometry influence the net transfer of individual elements between ecosystems. Here we develop a zero net transfer isocline (ZNTI) framework that integrates the relative mass and stoichiometry of fluxes into and out of an ecosystem. We then use case studies with amphibians and salmon to elucidate how life history, ontogenetic shifts in stoichiometry, and trophic interactions shape relative fluxes of nutrients between aquatic and terrestrial ecosystems. Because they increase in both size and Ca content from ova to metamorphs, amphibian life histories strongly bias them toward net Ca export into the terrestrial environment. Because amphibian biomass, C, P, and Ca ZNTIs do not overlap, there is no value of survivorship where the net flux of biomass, C, P, and Ca are simultaneously balanced between terrestrial and aquatic habitats. The degree of iteroparity and semelparity in salmon strongly affects both the magnitude of net biomass and P flux between riverine and marine environments. While the net direction of biomass flux generally remains strongly biased toward import into the riverine system, net P flux can reach net export into the marine environment because of increasing adult breeding survival leading to reduced mass and %P of what they deposit in rivers (e.g., ova vs. whole carcasses). These examples highlight how ontogenetic shifts in body size and stoichiometry result in asymmetric fluxes of elements and biomass that can lead to simultaneous net imports and exports of different elements within the same system. Furthermore, they demonstrate how changes in life-history characteristics and stage-specific survivorship can lead to changes in net elemental transport between ecosystems.

## Introduction

The movement of energy and nutrients between ecosystems has far-reaching impacts on the structure of food webs and their productivity (Polis et al., 1997). Both physical forces (e.g., wind, flooding) and motile organisms move nutrients and energy between ecosystem compartments (e.g., benthos and water column of a pond) and among ecosystems (DeAngelis, 1992; Vanni, 2002; Regester et al., 2006). The movement of organisms generates a unique type of material flux among ecosystems as its quantity and stoichiometry are subject to evolutionary, ecological, and physiological pressures and constraints. Furthermore, the movement of living biomass across ecosystem boundaries is governed by the movement ecology of the organisms themselves instead of being limited to only passive transport by physical forces.

Organisms move energy and nutrients among ecosystems through two main pathways, excreta and their individual bodies. Organisms with feeding migrations that move across ecosystem boundaries forage, excrete, and egest in different habitats, resulting in a strong directional flux out of the foraging environment and into the habitat where they deposit nutrients (e.g., sea birds; Anderson and Polis, 1999, hippopotomi; Subalusky et al., 2015). Inter-ecosystem fluxes of living biomass likewise have important effects on recipient systems (Helfield and Naimann, 2001, 2006; Marczak et al., 2007). Furthermore, several of these living energy and nutrient vectors have life cycles that obligatorily tie them to multiple habitats, creating reciprocal fluxes of biomass among those habitats (e.g., many insects, diadromous fishes, and amphibians). In these cases, anything that shapes the stoichiometry and or quantity of individuals moving between ecosystems may change the quantity and or quality of the flux itself.

Several factors can contribute to within species stoichiometric variation including local environmental differences and phenotypic variation (El-Sabaawi et al., 2012). In particular, when the relative contribution of body structures (e.g., phosphorus-rich bone) to whole body mass changes across ontogeny, so too does whole body stoichiometry (Sterner and Elser, 2002; Luhring, 2013; Boros et al., 2015; Stephens et al., 2016). Thus, organisms that simultaneously change in body composition and move between ecosystems change the stoichiometry of the biomass they bring with them. In these cases, even when the total biomass coming into and out of a system is equivalent, changes in the stoichiometry of the biomass coming in and going out of the system results in net import or export of individual elements (Luhring, 2013).

Though previous work has successfully cataloged the flow of materials among habitats and subsequent impacts on recipient ecosystems (e.g., Regester et al., 2006; Schriever et al., 2014; Capps et al., 2015), we still lack a conceptual framework to describe how the quantity and stoichiometry of organisms influence the net transfer of individual elements across habitat boundaries. Here we develop a framework integrating the quantity and stoichiometry of biomass movement between two systems.

We first develop the framework conceptually and then use four case studies to illustrate the use of this approach. In the first case study, we use this framework for amphibians with obligate aquatic and terrestrial life stages and show how changes to size and elemental composition between these stages alters the survival needed for net directional flux of biomass and different elements into or out of the aquatic system. In the second case study, we apply data from an amphibian mesocosm experiment to illustrate that the movement of individuals may cause biomass and different elements to vary in both the direction and magnitude of their net flux, and that trophic interactions that alter survivorship and life history alter the net influx and outflux of different elements. Third, we use Atlantic salmon to illustrate the effects of complete adult breeding mortality (semelparity) on biomass and P transfer between marine and riverine systems. Fourth, we develop the Atlantic salmon model to include partial adult breeding mortality (iteroparity) which allows for intermediate possibilities between the amphibian (breeding mortality absent) and semelparous salmon (100% breeding mortality) examples.

## Materials and methods

### The zero net transfer model

In our model there are two flux directions that occur between two ecosystems within a set time period (e.g., 1 lifecycle, 1 year). For simplicity, we will define terms in relation to a focal system and define fluxes as “in” (components moving into the focal system) and “out” (components moving out of the focal system). Because we are interested in the net direction of overall flux, we define the conditions needed for them to be equal and then explore conditions that cause net flux transfers to deviate from balanced conditions. Thus, we start with the assumption that Total Flux In = Total Flux Out, not because we expect the fluxes in to be equal to the fluxes out, as our results below will show, but because this is the most obvious reference point for assessing net import or export. We define the “in” flux as equal to the product of the number of components entering the system (*N*_*In*_) and their per capita mass (*Mass*_*In*_) (Equation 1). Likewise, the “out” flux is equal to the product of the number of components (*N*_*Out*_) and their per capita mass (*Mass*_*Out*_). Given an assumption that the flux in equals the flux out, we have:

(1) $$\begin{array}{l} N_{In}*\;Mass_{In}=N_{Out}*\;Mass_{Out} \end{array}$$

If we rewrite Equation (1) with *N* terms on the same side, we get an equation describing the inverse relationship between the relative number of individuals moving in each direction and their relative masses (Equation 2).

(2) $$\begin{array}{l} \frac{N_{Out}}{N_{In}}=\;\frac{Mass_{In}}{Mass_{Out}} \end{array}$$

**Figure 1:**
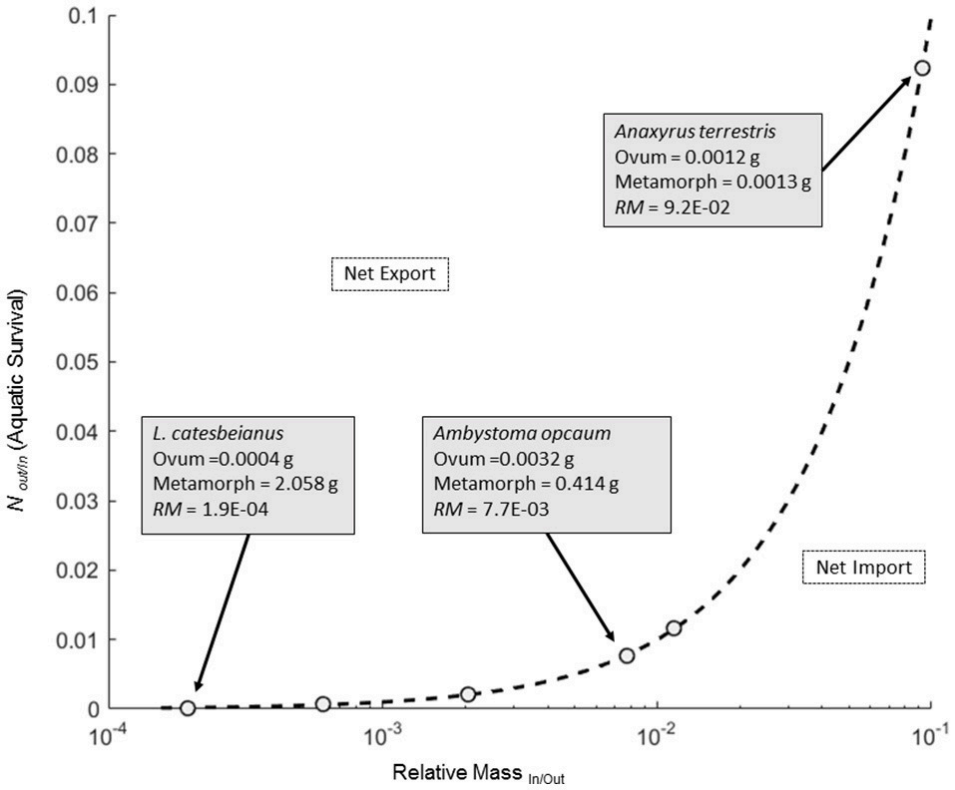
**Plot of the zero net transfer isocline (ZNTI; solid line) across a range of relative masses (***RM***) of ova and metamorphs for six amphibians**. Open circles illustrate the location of six *RM* values for amphibians from Table 1. Details for three data points are shown in boxes with an arrow pointing to their corresponding location on the isocline. Space below the ZNTI correspond to values of *RM* and *N*_Out/In_ where there is a net biomass transfer into the aquatic system, whereas space above the ZNTI corresponds to net biomass transport out of the aquatic system.

This equation can then be solved for values of mass and number where both sides are equal to each other, and we can use this solution as a zero net transfer reference isocline (ZNTI) to graphically depict expected patterns of biomass transport given equal fluxes in and out. Any deviations from this isocline tell us if a system is moving toward net import (values below the isocline) or export (values above the isocline) of biomass (see Figure 1 for example ZNTI).

To specify relative amounts of elements moving in each direction, we can adjust the biomass isocline equation (Equation 2) to include element-specific terms. *E*_In_ and *E*_Out_ are the proportions of each flux composed of a specific element “*E*.”

(3) $$\begin{array}{l} \frac{N_{Out}}{N_{In}}=\;\frac{Mass_{In}*E_{In}}{Mass_{Out}*\;E_{Out}} \end{array}$$

We simplify Equation (3) by defining the relative per capita mass of the “in” flux to the “out” flux as RM_In/Out_ and the relative elemental composition of the “in” flux to the “out” flux as RE_In/Out_. Rewriting Equation (3) with these definitions, we get:

(4) NOutNIn=RMInOut*REInOut

### Case study 1: shifts in amphibian stoichiometry across ontogeny

Amphibians with complex life histories move biomass and nutrients from terrestrial into aquatic systems in the form of their ova and transport biomass and nutrients from aquatic systems into terrestrial systems in the form of their metamorphosing juveniles (hereafter “metamorphs”; Regester et al., 2006). We set the aquatic system as the focal system whereby ova are the “in” flux and metamorphs are the “out” flux. Substituting flux stages for “in” and “out” we get the biomass ZNTI for this system:

(5) NMetamorphsNOva=RMOvaMetamorphs*REOvaMetamorphs

The left hand side of Equation (5) describes the proportion of ova surviving to metamorphosis (larval survival); the RM term describes the relative size of an ovum to a metamorph; and the RE term describes the relative elemental composition of an ovum relative to a metamorph.

We used dry mass and elemental data collected for ova and metamorphs of six amphibian species to demonstrate how changes in body size and stoichiometry across ontogeny affect the net movement of biomass and elements between terrestrial and aquatic ecosystems (see Luhring, 2013 for details). Briefly, samples of each species were collected from Aiken, South Carolina and vacuum dried (Labconco Freezone vacuum dryer) prior to being homogenized for elemental analyses. C and N were analyzed at the MBL Stable Isotope Laboratory, Woods Hole, MA with a Europa ANCA-SL elemental analyzer. All other elements were analyzed at the University of Georgia's Soil, Plant, and Water Laboratory through microwave assisted digestion (EPA method 3052) followed by axially viewed ICP-AES (EPA method 6010b).

We calculated metamorph masses of the six species in two ways to account for differences in the magnitude of variation seen in the larger species. For the largest four species (*Ambystoma opacum, Ambystoma talpoideum, Lithobate catesbeianus, Lithobates sphenocephalus*), we use the midpoint between the largest and smallest individuals. For the smallest two species (*Anaxyrus terrestris* and *Scaphiopus holbrookii*), we used composite samples of 15–30 individuals to estimate an average body size (i.e., multiple individuals dried in groups, weighed, and total mass divided by number of individuals). Composite samples also were used to approximate individual ovum dry mass. Elemental data for ova were derived from two different composite samples. For the four amphibians metamorphosing at larger sizes we averaged elemental data from large individuals and composites of small individuals (two samples in total). Metamorph elemental data from the two smallest species are derived solely from composite samples.

These data were then used to calculate *RM*'s and *RE*'s for each species. For each species' *RM*, we solve for values of larval survival that results in a balanced transfer of biomass. We then use an average amphibian *RE* to create ZNTI's for three elements (P, C, and Ca) to illustrate how the locations of element-specific ZNTIs may not line up with the biomass ZNTI. What this shows is that any given level of survivorship will likely not balance the import and export of different elements or biomass at the same time.

### Case study 2: applying experimental amphibian data to isoclines

Amphibian body size at metamorphosis, survival, and thus biomass export is affected by a myriad of factors (Semlitsch and Caldwell, 1982; Morin, 1986; Earl et al., 2011). We estimated *RM*'s and survivorship from a mesocosm experiment (see Luhring, 2013 for details) which manipulated light, competition, and predation on three anuran species representing a continuum of *RM*'s: *Anaxyrus americanus* (RM = 1/10), *Hyla versicolor* (1/150), *L. sphenocephalus* (1/185). Briefly, each species was raised in replicated 1000-L mesocosms either alone (single-species), with all three anuran species together (competition treatment), or all three anuran species and caudate predators (competition + predation treatment). All of these treatments were crossed with 2 light levels: high (77% of ambient) and low (27% of ambient) light. Anuran larvae were removed from the mesocosms upon metamorphosis, measured and weighed (wet mass in g). A subset from each species were subsequently measured for dry mass to create species-specific wet mass to dry mass conversion rates for the experiment (*A. americanus*: 0.113, *H. versicolor*: 0.143, and *L. sphenocephalus*: 0.149). For each species, we used individual ova dry mass from batches of ova for *L. sphenocephalus* (0.0013 g) or for congeneric species as necessary (*A. terrestris*: 0.0012 g, *H. cinerea*: 0.0006 g). Mesocosm average metamorph dry masses were derived by converting average metamorph wet mass to dry mass by their species- or genera-specific wet to dry mass conversion. We then use average mesocosm *RM* and realized larval survival rate to graphically depict the effects of light, competition and predation on net movement of biomass and Ca. Plots of biomass isoclines represent x-fold isoclines for each element where x is equal to the inverse of the product of *RE* and *RM*. For example, the ZNTI for biomass is a 1-fold export (where the ratio of biomass export to biomass import is 1), and a 10x NTI for biomass is a 10-fold export. For a species with an *RE*_*Ca*_ of 1/45, 1x and 10x biomass isoclines would be equal to 45-fold, and 450-fold net transfer isoclines of Ca, respectively.

### Case study 3: complete breeding mortality in Semelparous Salmonids

Salmonids move massive amounts of biomass between marine and riverine systems every year and the resultant effects of this flux on recipient systems (e.g., Janetski et al., 2009) and the net flow of nutrients among them are of particular interest (e.g., Moore et al., 2007; Ebel et al., 2015). Semelparous salmonids flux biomass and nutrients from marine into riverine systems in the form of their entire body mass (including gonads) and flux biomass and nutrients from riverine systems into marine systems in the form of their juveniles (hereafter “smolts”; e.g., Gende et al., 2002; Schindler et al., 2003; Ebel et al., 2015). We set the river as the focal system whereby adult salmon are the “in” flux and smolt are the “out” flux. Substituting flux stages for “in” and “out” we get:

(6) NSmoltsNAdults=RMAdultsSmolts∗REAdultsSmolts

The left hand side in Equation (6) describes the per capita conversion of in-migrating adults into out-migrating smolts (per capita net recruitment), the RM term describes the relative size of an adult to a smolt, and the RE term describes the relative elemental composition of an adult relative to a smolt. We used wet mass and P content collected for Atlantic salmon from three river systems (Ebel et al., 2015) to demonstrate how size and elemental asymmetries between smolt and adult salmon of different rivers affect net biomass and P flux between marine and riverine systems. We solve for *RM* and *RE*_*P*_ using the same approach as described in the previous examples. This data is then used in a subsequent adaptation of our model for incomplete breeding mortality (see Case Study 4: Partial Breeding Mortality in Salmonids).

### Case study 4: partial breeding mortality in salmonids

Although many species of salmonids are semelparous (100% adult mortality after breeding), some are at least partially iteroparous and spend part of their post-breeding recovery time in the river in which they breed. Incomplete breeding mortality results in an additional life stage (hereafter “kelts”) moving biomass and nutrients out of the river which is partially offset by what it deposits while in the river (gametes, egested, and excreted materials). Using the same source of data from Section Case Study 3: Complete Breeding Mortality in Semelparous Salmonids, we adapt Equation (1) to include partial mortality of adults and bi-directional movement of materials by overwintering kelts. Because the mass and stoichiometry of kelts leaving the river is different than that of adults entering the river, the *Mass*_In_ term has to account for changes in mass, elements, and the mortality of adults. The adult mass that enters the system and stays in is the fraction of those adults that die times the mass and elemental composition of adults (*D* * *M*_*A*_ * *E*_*A*_), with terms subscripted *A* for adult. Likewise, the adult mass that leaves the system is the fraction of the adults that live times the mass and elemental composition of kelts [(1−*D*) * (*M*_*A*_ * *E*_*A*_ − *M*_*K*_ * *E*_*K*_)], with terms subscripted *K* for kelt. Substituting these expressions into Equation (1), and subscripting smolt terms with an S, we get:

(7) $$\begin{array}{l} N_{A}(D*M_{A}*E_{A}+(1-D)*\left(M_{A}*E_{A}-M_{K}*E_{K}\right))\\ \;=N_{S}*M_{S}*E_{S} \end{array}$$

This simplifies to the elemental ZNTI for salmon systems:

(8) NsNA=D∗RMAS∗REAS          +(1−D)∗(MA∗EA−MK∗EK)MS∗ES 

that like the previous ZNTIs, relates the transformation of individuals while in the system to the changes in individual mass and elemental composition while in the system.

We use the same wet mass and P content collected for Atlantic salmon from the three river systems as in the previous example (Section Case Study 3: Complete Breeding Mortality in Semelparous Salmonids; Ebel et al., 2015) to demonstrate how different levels of adult breeding mortality (*D*) affect net biomass and P fluxes. This model (Equation 8) thus offers an intermediate between the amphibian example (Section Case Study 1: Shifts in Amphibian Stoichiometry Across Ontogeny, Equation 5) and the semelparous salmon example (Section Case Study 3: Complete Breeding Mortality in Semelparous Salmonids, Equation 6) which are derived assuming either 0 or 100% breeding mortality respectively. When *D* is 1, complete breeding mortality occurs and Equation (8) becomes identical to Equation (6). When *D* is 0, it effectively becomes Equation (5) wherein the elemental content of an adult coming in minus its elemental content going out is the contribution to the “in” habitat (e.g., ova).

## Results and discussion

### Case study 1: shifts in amphibian stoichiometry across ontogeny

The amphibians we considered varied considerably in relative sizes of ova to metamorphs, with the *RM*_*In*/*Out*_ ranging from 1/5145 to 1/11 (Figure 1; Table 1). Because a ZNTI is essentially a 1:1 line, these values stipulate the survival required to balance the transfer of mass into and out of the system. Although survivorship to metamorphosis is highly variable in natural amphibian populations, we can get an idea of where different species fall along the spectrum of export vs. import potential. Species with small ova and a large size at metamorphosis have the smallest *RM*_*In*/*Out*_ and, subsequently, the lowest survival required for a zero net transfer of biomass. Conversely, species with the largest *RM*_*In*/*Out*_ recorded in this study (*A. terrestris; RM*_*In*/*Out*_ = 1/11) had the smallest size at metamorphosis (0.001 g dry mass) and the highest survival required for zero net transfer of biomass (1 in 11 ova surviving to metamorphosis). Because both ovum size and size at metamorphosis determine *RM*_*In*/*Out*_, species with similar *RM*_*In*/*Out*_'s such as *S. holbrookii* and *A. opacum* (1/86.7 vs. 1/129.4) may differ both in ova size (2.6-fold difference) and size at metamorphosis (4-fold difference) yet still require the same survival to balance mass transfers (Table 1).

**Table 1:** Summary of ova and metamorph size and elemental composition for 6 amphibian species with larval survivorship values (boldfaced) where biomass flux (***RM***) and element-specific flux (RM^*^RE) are balanced between terrestrial and aquatic systems.

**Species**	**Stage**	N^*^	**Mean mass**		C^**^	N^**^	Ca^***^	P^***^	S^***^	**C:Ca**
*Ambystoma opacum*	Ova	695	0.0032		50.56	10.22	475	11,976	5429.5	1064.5
	Meta	15	0.414		45.80	10.80	27,342	17325.5	6675.5	16.7
		***RM*** =	**7.7E-03**	***RE*** =	1.10	0.95	0.02	0.69	0.81	
		RM^*^RE =			**8.53E-03**	**7.31E-03**	**1.34E-04**	**5.34E-03**	**6.29E-03**	
*Ambystoma talpoideum*	Ova	2,670	0.0011		49.55	10.50	603	13048.5	5,818	821.7
	Meta	5	1.805		43.36	9.02	23204.5	15402	6449.5	18.7
		***RM*** =	**6.1E-04**	***RE*** =	1.14	1.16	0.03	0.85	0.90	
		RM^*^RE =			**6.96E-04**	**7.10E-04**	**1.58E-05**	**5.16E-04**	**5.50E-04**	
*Anaxyrus terrestris*	Ova	3,444	0.0012		50.59	9.96	577	12445.5	6209.5	876.8
	Meta	306	0.013		37.31	9.73	25,691	16,477	5,863	14.5
		***RM*** =	**9.2E-02**	***RE*** =	1.36	1.02	0.02	0.76	1.06	
		RM^*^RE =			**1.25E-01**	**9.45E-02**	**2.07E-03**	**6.97E-02**	**9.78E-02**	
*Lithobates catesbeianus*	Ova	31,806	0.0004		51.56	11.35	1,371	9332.5	7132.5	376.1
	Meta	2	2.058		45.76	9.95	32230.5	18544.5	5,467	14.2
		***RM*** =	**1.9E-04**	***RE*** =	1.13	1.14	0.04	0.50	1.30	
		RM^*^RE =			**2.19E-04**	**2.22E-04**	**8.27E-06**	**9.78E-05**	**2.54E-04**	
*Lithobates sphenocephalus*	Ova	4,424	0.0013		51.17	10.16	556.5	11,079	6335.5	919.5
	Meta	4	0.638		41.89	9.72	25,501	14,211	5685.5	16.4
		***RM*** =	**2.0E-03**	***RE*** =	1.22	1.05	0.02	0.78	1.11	
		RM^*^RE =			**2.49E-03**	**2.13E-03**	**4.45E-05**	**1.59E-03**	**2.27E-03**	
*Schaphiopus holbrookii*	Ova	2,400	0.0012		50.25	9.77	1,373	11949.5	6,376	366.0
	Meta	13	0.104		48.75	8.98	24,054	11,938	4,715	20.3
		***RM*** =	**1.2E-02**	***RE*** =	1.03	1.09	0.06	1.00	1.35	
		RM^*^RE =			**1.19E-02**	**1.26E-02**	**6.59E-04**	**1.15E-02**	**1.56E-02**	

*RM, relative mass (ovum mass/metamorph mass). RE, Relative elemental composition (ovum element concentration/ metamorph elemental concentration). Mass is in g dry mass*.

* *Total individuals in all composite samples*.

** *Elemental content as percent of dry mass*.

*** *Elemental content as mg kg^−1^ of dry mass*.

**Figure 2:**
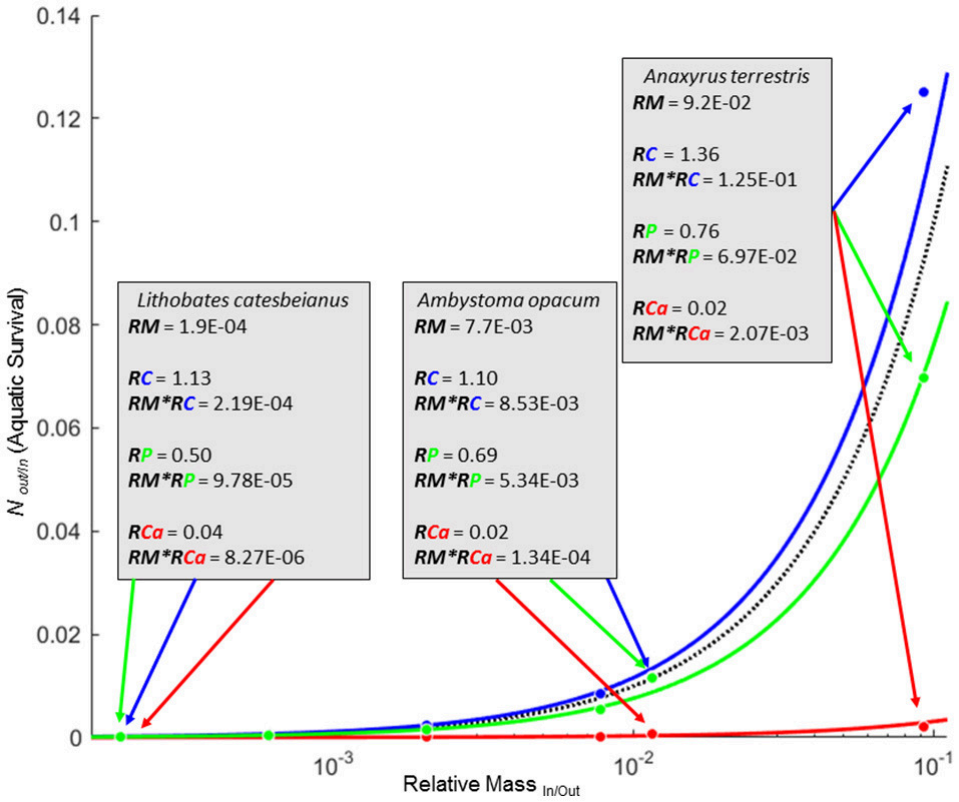
**Zero net transfer isoclines (ZNTI) for biomass (black), C (solid blue), P (solid green), and Ca (solid red) for 6 amphibian species where ***P*** corresponds to balanced flux between terrestrial and aquatic systems for biomass, C, or Ca**. Isoclines for C and Ca depict the average *RE* for C and Ca for the six amphibians. Details for three species are shown in boxes with arrows pointing to their corresponding location on the isoclines. Regions between lines represent simultaneous import and export of two different materials. For example, the region between biomass and Ca isoclines represent values at which there would be an import of biomass and an export of Ca.

Whenever elemental composition changes with life history stages of individuals entering or leaving a focal system (RE ≠ 1), the net flux of biomass and that element cannot be equivalent. For our set of amphibians, there was considerable asymmetry in the transport of biomass and elements into and out of a pond. *RE*_*In*/*Out*_ net direction and magnitude for each element was generally consistent across amphibian species, but varied widely across elements (Table 1). *RE*_*In*/*Out*_ ranged from a high of 1.36/1 for *RC*_*In*/*Out*_ (*A. terrestris*) to a low of 1/57.56 for *RCa*_*In*/*Out*_ (*A. opacum*; Table 1). The six species in this study all had at least an order of magnitude decrease in C:Ca ratio from ova to metamorphosis driven by both a decrease in C and a 17- to 57-fold increase in Ca (Table 1). Of all the elements, Ca was the most strongly asymmetric between ova and metamorphs (average *RCa*_*In*/*Out*_ = 1/37.9). The combined strong asymmetries in both *RCa* and *RM* of amphibians meant that most species were strongly biased toward exporting Ca even at levels with biomass or P import (Figure 2; RE^*^RM in Table 1).

Similar to how fluxes of elements with RE ≠ 1 cannot be simultaneously balanced with biomass fluxes, fluxes of elements with differing *RE*'s cannot be simultaneously balanced with each other. For any given element “Y,” there are two scenarios when the incoming and outgoing fluxes have different “Y” values (*RY*_*In*/*Out*_ ≠ 1): (1) *P*_0,*Y*_ isocline is above the *P*_0,Biomass_ isocline (*RY*_*In*/*Out*_ > 1; bias toward import), or (2) *P*_0,*Y*_ isocline is below the *P*_0,Biomass_ isocline (*RY*_*In*/*Out*_ < 1; bias toward export). When elemental *P*_0_'s are situated above and below the *P*_0*Biomass*_ isocline (e.g., C and Ca in Figure 2), there exist several possible outcomes for element-specific transfer between systems. For example, any point along the *P*_0,Biomass_ isocline in the amphibian example (Figure 2) would correspond with a net import of C and a simultaneous export of Ca. However, any point along the *P*_0_,_Ca_ isocline where Ca movement between the systems is balanced would correspond to a strong import of biomass and C. Values not falling directly on any isoclines demonstrate simultaneous inequalities in net movement for all materials considered (i.e., biomass, C, P, and Ca in Figure 2).

### Case study 2: applying experimental data to isoclines

**Figure 3:**
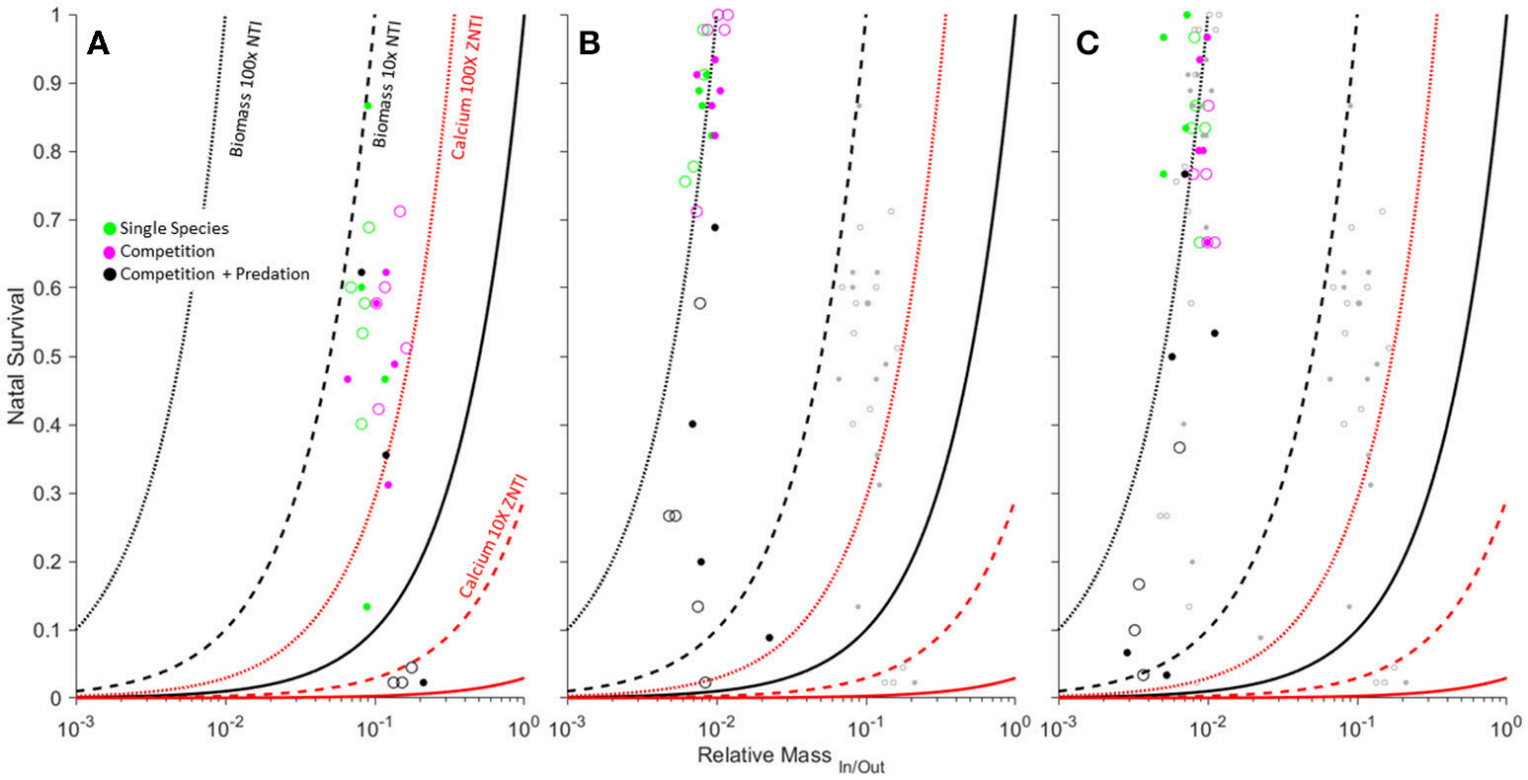
**Effects of competition, predation and light levels (low [open] vs. high [filled]) on biomass and calcium transfer magnitude for ***Anaxyrus americanus*** (A)**, *Hyla versicolor* **(B)**, and *Lithobates sphenocephalus* **(C)**. Data points represent mesocosm average relative mass of ova/metamorphs (*RM*) and larval survivorship (*N*_*Out*/*In*_) from a previous study (Luhring, 2013). Zero net transfer isoclines for biomass (solid black), and Ca (solid red) and their 10x and 100x net transfer isoclines (dashed and dotted NTI's) are shown for reference. Each panel includes the data from panels to their left as gray dots for reference.

Changes in average body size at metamorphosis and larval survival resulting from predation readily moved mesocosms across biomass and elemental isoclines (Figure 3). The smallest species (by metamorph size) was most readily drawn below the biomass ZNTI through predation, but remained above the Ca ZNTI unless there was 0 survivorship (Figure 3). *A. americanus* failed to metamorphose from 6 of 12 predator mesocosms, in contrast to *H. versicolor* and *L. sphenocephalus* which both failed to metamorphose from 3 of 12 predator mesocosms. In these cases, our gape-limited predators appeared to have a relatively stronger ability to force the net flux of the smaller species into a state of total import (i.e., a complete biomass and nutrient sink).

Although changes to either body size or survivorship would have predictable effects on net transfers of biomass, they are not always independent of each other. This is because body size at metamorphosis results from the interaction of a multitude of environmental and evolutionary factors (Werner, 1986). Links between body size at metamorphosis and aquatic survival thus change the rates at which changes in survival may shift systems between states of net import or export. The causes of linked *RM*_*In*/*Out*_ (as a function of changing size at metamorphosis) and survival in our systems are well-known and provide an opportunity to understand how these phenomena affect the movement of biomass and nutrients across ecosystems.

The smallest species, *A. americanus*, demonstrates an Allee effect whereupon size at metamorphosis is highest at an intermediate density (our stocking density), but decreases with a decrease in population density (Wilbur, 1977). Our mesocosms demonstrated a similar pattern for *A. americanus* whereby predation lowered population density of *A. americanus* in our mesocosms and reduced the sizes of the few successfully metamorphosing juveniles. Predation thus appeared to accelerate *A. americanus* toward net import by simultaneously increasing the required survivorship for balanced flux (through increased *RM*_*In*/*Out*_) while decreasing survivorship. Predation can also lead to a thinning effect whereby predators decrease population density and thus intra or interspecific competition, leading to larger sizes at metamorphosis when survival decreases (Wilbur et al., 1983). This pattern was most apparent in our largest species, which may be heavily predated at smaller sizes but escape gape-limited predators at larger sizes. In their case, the largest metamorphs and thus smallest mean *RM*_*In*/*Out*_ values came from mesocosms with the lowest aquatic survival rates (Figure 3). Although predation decreases survivorship in the larger species, the ability of predators to draw the system into a net import is partially offset by a simultaneous decrease in *RM* caused by a larger size at metamorphosis.

Because elemental transfer isoclines are directly linked to biomass isoclines through Equation (5), any isocline that is a multiple of the biomass ZNTI can be readily interpreted for a given element by multiplying the biomass NTI by the inverse of the *RE*_*In*/*Out*_ for the element of interest. This allows the use of additional biomass NTI's (10x NTI, 100x NTI, etc.) that can be used as reference contours to depict the relative magnitude of both biomass and elemental import or export. For example, a 10x NTI and 100x NTI indicate the 10- and 100-fold relative export of biomass (Figure 3). In the case of *L. sphenocephalus* where *RCa*_*In*/*Out*_ = 1/45.82 and *RC*_*In*/*Out*_ = 1/0.82 (Table 1), the 10x NTI would correspond to a 458.2x NTI for Ca, and a 8.2x NTI for C. The 100x NTI where many of the *L. sphenocephala* mesocosms are clustered (right panel of Figure 3) would thus correspond with a 4582x NTI for Ca and a 82x NTI for C.

### Case study 3: complete breeding mortality in Semelparous Salmonids

**Figure 4:**
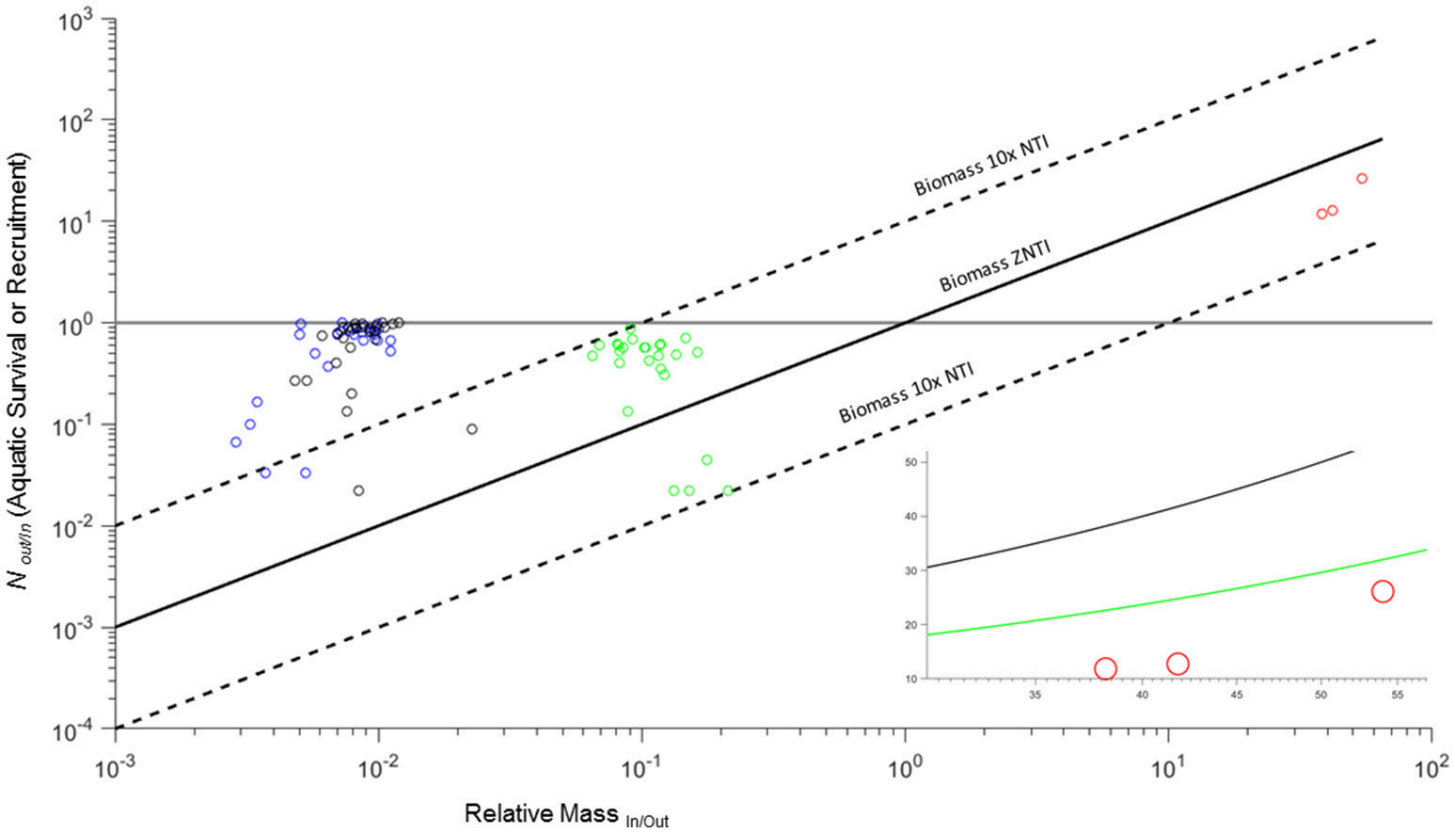
**Zero net transfer and 10x net transfer biomass isoclines for ***RM***_***In***/***Out***_ and ***N***_***Out***/*In*_ for amphibian mesocosms (blue, black, and green: ***L***. ***sphenocephalus***, ***H. versicolor***, and ***A. americanus***) and Atlantic salmon (red: assuming complete breeding mortality)**. Horizontal gray line indicates the upper limit of *N*_*Out*/*In*_ for aquatic survival for amphibian larvae (cannot exceed 1). Inset image shows salmon data against the biomass ZNTI (black line) and P ZNTI (green line: derived from average smolt and adult P across three populations).

As with amphibians, two life stages of salmon move biomass between ecosystems. However, semelparous salmon are different in two key ways. First, breeding adults deposit their entire body mass (body and gametes) in the “in” system (river) when they die. Second, while the conversion of N_*In*/*Out*_ (larval survival) for amphibians has an upper limit of 1 (solid horizontal line in Figure 4), N_*In*/*Out*_ for salmon is a measure of net recruitment of smolts per breeding adult and has a much higher ceiling. These combined factors extend the salmon biomass ZNTI farther to the right of the amphibians while still allowing for potential import or export (Figure 4).

Salmon in the three rivers examined had net recruitment rates (*N*_*Out*/*In*_) of 11.90–26.15 (Table 2). If we assume that spawning adults in these rivers experience complete mortality (Equation 6), then net biomass flux would be into the river as all recruitment rates were below the ZNTI (Figure 4; Table 2). To investigate net P flux, we incorporate P composition for adults and smolts. Because in-migrating adults are lower in P than out-migating smolts (*RP*_*In.Out*_ < 1), they lower the P isocline relative to the biomass isocline (inset Figure 4). Thus, although adults represent a higher per capita biomass flux, their lower P content reduces the amount of relatively P-rich smolt biomass that has to exit the system in order for P flux to be balanced. None of the rivers had the net recruitment needed to reach their biomass ZNTIs (*N*_*Out*/*In*_*/RM*_*In*/*Out*_) or net recruitment need to reach their P ZNTIs (*N*_*Out*/*In*_/RM*RP). However, one river (Conne; Table 2) reached 87% of the recruitment needed to reach its P ZNTI (Table 2). This case study demonstrates the outcomes of complete breeding mortality (semelparity) on cross-ecosystem fluxes of biomass and elements. However, for many diadromous fishes, some fraction of adults survive spawning events and exit the breeding system (e.g., iteroparous species). Below, we will see how these outcomes shift after incorporating partial breeding mortality in the same system.

**Table 2:** **Summary of Atlantic salmon adult, smolt, and kelt size and P content by river from Ebel et al. (**2015**) with derived model parameters (boldface)**.

**River**	**Stage**	Wet mass (g)^*^	%P^**^	**RM_*In*/*Out*_**	**RP_*In*/*Out*_**	RM ^*^ RP	***N_*out*_***	***N_*in*_***	***N_*out*/*In*_***
Cambellton	Adult	1,910	0.38	**38.2**	**0.66**	**25.03**	40,146	3,375	**11.90**
	Smolt	50	0.58						
	Kelt	1331.4	0.5			***N_*out*/*In*_/RM_*In*/*Out*_***			**0.31**
	Adult-Kelt	578.6	0.10			N_*out*/*In*_/(RM^*^RP)			**0.48**
Conne	Adult	1,620	0.36	**54**	**0.55**	**29.91**	67,209	2,570	**26.15**
	Smolt	30	0.65						
	Kelt	730	0.53			***N_*out*/*In*_/RM_*In*/*Out*_***			**0.48**
	Adult-Kelt	890	0.22			N_*out*/*In*_/(RM^*^RP)			**0.87**
Western Arm	Adult	2,090	0.37	**41.8**	**0.57**	**23.79**	15,756	1,234	**12.77**
	Smolt	50	0.65						
	Kelt	826.9	0.58			***N_*out*/*In*_/RM_*In*/*Out*_***			**0.31**
	Adult-Kelt	1263.1	0.23			N_*out*/*In*_/(RM^*^RP)			**0.54**

Adult-Kelt quantifies mass loss and the %P of that loss for adults surviving the breeding season. N_Out/In_/RM and N_Out/In_/RM ^*^RP are the amount of recruitment observed relative to that needed for zero net transfer of biomass and P (respectively) between river and marine systems.

* *Adult mass is a weighted mean of wet mass (g) weighted by number of adults in two size classes*.

** *%P is on a wet mass basis*.

### Case study 4: partial breeding mortality in salmonids

Many reciprocal fluxes resulting from linked life stages may fall between the extremes of complete breeding mortality (semelparity) and complete survival of breeding adults (e.g., an implicit assumption of the amphibian mesocosm example). Equation (8) describes the intermediate case of partial breeding mortality that simplifies into Equation (6) when *D* = 1 (semelparity) and Equation (4) when *D* = 0 (where eggs are deposited by females who then leave the system). The change from deposition of an adult carcass to partial deposition of adult biomass (“Adult-Kelt” in Table 2) results in a net per capita reduction of biomass deposited by adults. Increases in breeding mortality (*D*) increase the per capita contribution of adults to the “in” fluxes of biomass and raise the recruitment rate required to reach the biomass ZNTI. Decreases in *D* decrease the per capita contribution of adults to the “in” fluxes of biomass and lower recruitment rates required to reach the biomass ZNTI. Although adults are lower in P content than kelts, the partial contributions of adults to rivers resulting from breeding activity and overwintering are even lower in biomass and %P content (“Adult-Kelt” in Table 2). Because P in this partial deposition is lower than that of adult carcasses, the total amount of P deposited per adult decreases at a faster rate than biomass with increased adult breeding survival (1 − *D*).

**Figure 5:**
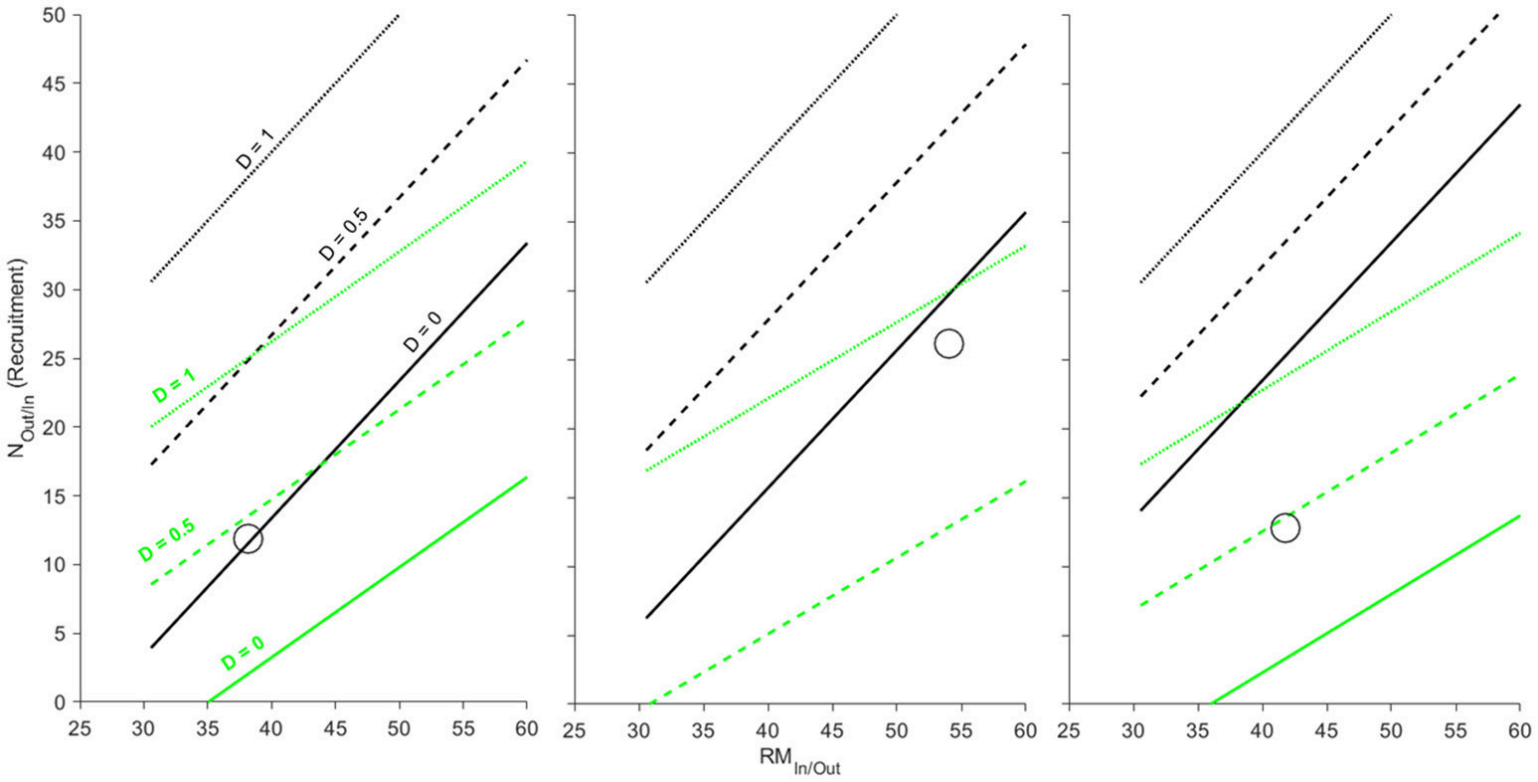
**Zero net transfer isoclines for biomass (black) and P (green) for three populations of Atlantic salmon (from left to right: Cambellton, Conne, and Western Arm; data from Ebel et al., **2015**)**. ZNTI's are shown for varying levels of breeding mortality (*D* = 0, 0.5, 1) and are derived for population-specific differences in stage-specific size, %P, spawning run size (*N*_*In*_) and smolt production (*N*_*Out*_). Only when 100% of adults survive and become outmigrating kelts in Cambellton (*D* = 0) would a net biomass export into the marine environment occur (above black line). All populations show potential for P import or export based on varying levels of breeding mortality (between green lines for *D* = 0 and *D* = 1).

Shifting from complete breeding mortality to partial or complete survival of breeding adults changes the net biomass or P flux between rivers and marine systems. Scenarios where adult breeding mortality is 100% (*D* = 1; dotted black and green lines in Figure 5) are identical to that of the previous example (Figure 4; Section Case Study 3: Complete Breeding Mortality in Semelparous Salmonids) and serve as a reference. Even with 100% breeding survival (*D* = 0; solid lines in Figure 5), biomass flux generally remained biased toward import with only the Cambellton River showing a minor net export of biomass (Figure 5 left panel). However, P flux among systems was more variable. As opposed to net biomass flux, P was much more likely to transition to a net export from rivers to marine systems with increasing levels of adult survival (Figure 5). All rivers showed net P export when 100% of adults survived and were close to or above P ZNTIs when adult survival was 50% (Figure 5). Thus, while biomass in the salmon examples are strongly biased toward net import into rivers, partial breeding survival can transition these systems into simultaneous net fluxes of P out of and biomass into rivers.

## Conclusions

### How stoichiometry influences net fluxes of elements between ecosystems

When inter-ecosystem fluxes are carried out by different life stages of a single organism, predictable asymmetries in net biomass and elemental fluxes arise through changes in body size and ontogenetic changes in stoichiometry. For example, large shifts in both body size and C:Ca from ovum to metamorphosis create large size and elemental imbalances in amphibian derived biomass fluxes between aquatic and terrestrial ecosystems. Aquatic survival is generally low in amphibians (Herreid and Kinney, 1966; Calef, 1973; Shoop, 1974; Semlitsch, 1987; Regester et al., 2006), which may cause many species with smaller metamorphs relative to ova size (and thus larger RM's) to generally be net importers of biomass. However, because of the large increase in Ca from ova to metamorph, nearly all species of amphibians are also typically net exporters of Ca from wetlands, barring complete breeding failure. Less drastic changes to individual size and survivorship would be needed to switch elements with *RE*'s closer to 1 between net import and export.

### Future directions

We present a framework that can be adapted to include more complicated eco-evolutionary dynamics and their effects on inter-ecosystem fluxes. For example, relationships between survivorship and body size (e.g., thinning, Allee effects; Figure 3) and their subsequent effects on fluxes can be characterized by their effects on the slope of survivorship vs. relative mass. Furthermore, because stoichiometry changes with body size among and within species, the assumption that an *RE* value would be static across values of *RM* may not hold for all models. Although these initial models successfully describe the effects of asymmetries in body size and stoichiometry on reciprocal flux dynamics, additional modifications that reflect increasingly complicated dynamics (e.g., size and stoichiometry) may reveal additional insights.

The integration of the ZNTI approach into food web ecology would be a logical and important next step. While our models explicitly incorporate the effects of life stage asymmetries in size and stoichiometry on net transfer magnitude and direction, they do not yet integrate these asymmetries into food web dynamics (e.g., the merged ecosystem and food web approach advocated by Marcarelli et al., 2011). Relative composition, magnitude, and amount of available resources in incoming fluxes vs. existing resources determine the effects that incoming fluxes have on recipient ecosystems (Marczak et al., 2007; Marcarelli et al., 2011). Thus, the same stage-specific changes in size or stoichiometry in our models that determine net flux magnitude and direction will also determine their effects on recipient systems.

### Generalities of the isocline approach

Our modeling and isocline approach is readily applicable for a variety of systems for determining the effects of processes (e.g., per capita recruitment, larval survival) and properties of the fluxes (e.g., relative sizes and elemental composition) in changing net flux direction and magnitude between ecosystems. Because conversion rates of in to out (larval survival, smolt recruitment per adult) and their relative sizes and elemental composition are readily estimable or available in the literature for many systems, initial models can be used to estimate the relative sensitivities of flux direction and magnitude to changes in these processes and properties *a priori*. Gathering the data for individual flux components to estimate whether a system experiences a net import or export within or across seasons is highly time and resource intensive (e.g., Regester et al., 2006; Ebel et al., 2015). The details garnered from such studies are essential to understand variation in contributions of individual-level fluxes to between-ecosystem fluxes. However, a general model which can account for the wide disparity in processes that link reciprocal fluxes (e.g., larval survival, recruitment) to each other as well as their relative differences in size and elemental composition is required to begin synthesizing these processes across time and spatial scales.

## Ethics statement

This study was carried out in accordance with the University of Missouri's Animal Care and Use Committee under permit MU ACUC 6144, 7403.

## Author contributions

TL, JD, and RS contributed to the conception of the work and revised various drafts of previous versions. TL and JD wrote the final version of the manuscript.

## Funding

TL was funded by the University of Nebraska's Population Biology Program of Excellence. Postdoctoral Fellowship and the University of Missouri's Life Sciences and Trans-World Airlines Fellowships.

### Conflict of interest statement

The authors declare that the research was conducted in the absence of any commercial or financial relationships that could be construed as a potential conflict of interest.
